# Eosinophil biology from the standpoint of metabolism: implications for metabolic disorders and asthma

**DOI:** 10.1093/jleuko/qiae100

**Published:** 2024-05-03

**Authors:** Nana-Fatima Haruna, Sergejs Berdnikovs, Zhenying Nie

**Affiliations:** Division of Allergy and Immunology, Feinberg School of Medicine, Northwestern University, 240 East Huron, McGaw M309, Chicago, IL 60611, United States; Division of Allergy and Immunology, Feinberg School of Medicine, Northwestern University, 240 East Huron, McGaw M309, Chicago, IL 60611, United States; Division of Pulmonary and Critical Care Medicine, Oregon Health & Science University, 3181 SW Sam Jackson Park Rd, Portland, OR 97239, United States

**Keywords:** adipocytes, asthma, eosinophils, metabolism, obesity

## Abstract

Eosinophils, recognized for their immune and remodeling functions and participation in allergic inflammation, have recently garnered attention due to their impact on host metabolism, especially in the regulation of adipose tissue. Eosinophils are now known for their role in adipocyte beiging, adipokine secretion, and adipose tissue inflammation. This intricate interaction involves complex immune and metabolic processes, carrying significant implications for systemic metabolic health. Importantly, the interplay between eosinophils and adipocytes is bidirectional, revealing the dynamic nature of the immune–metabolic axis in adipose tissue. While the homeostatic regulatory role of eosinophils in adipose tissue is appreciated, this relationship in the context of obesity or allergic inflammation is much less understood. Mechanistic details of eosinophil–adipose interactions, especially the direct regulation of adipocytes by eosinophils, are also lacking. Another poorly understood aspect is the metabolism of the eosinophils themselves, encompassing metabolic shifts during eosinophil subset transitions in different tissue microenvironments, along with potential effects of host metabolism on the programming of eosinophil hematopoiesis and the resulting plasticity. This review consolidates recent research in this emerging and fascinating frontier of eosinophil investigation, identifying unexplored areas and presenting innovative perspectives on eosinophil biology in the context of metabolic disorders and associated health conditions, including asthma.

## Teaser

The complex interplay between eosinophils and adipocytes shapes metabolic homeostasis, but its dysregulation may contribute to metabolic conditions.

## 1. Introduction

Eosinophils are multifaceted immune cells renowned for their integral role in the immune pathology of asthma and have long captivated researchers and clinicians due to their intricate involvement in the pathogenesis of asthma and other allergic diseases.^1–4^ Despite the predominant focus of the eosinophil research on the asthmatic airways, a growing body of evidence highlights another arena where eosinophils exert their influence—adipose tissue. Adipose tissue, traditionally considered an energy reservoir, is now acknowledged as an active endocrine organ capable of secreting numerous bioactive molecules, including adipokines, chemokines, and cytokines.^5,6^ This revelation has expanded our understanding of adipose tissue far beyond its energy storage function, implicating it in diverse physiological and pathological processes. Notably, this includes its intricate interplay with the immune system, where immune cells such as eosinophils may exert unexpected effects on both adipose tissue function and systemic metabolism. The presence of eosinophils in adipose tissue creates a unique microenvironment where immune cells meet metabolic cells.^7–9^ Although the precise mechanisms driving eosinophil recruitment and retention in adipose tissue are still under investigation, it is clear that adipocytes and adipose-resident immune cells, including eosinophils, engage in a complex interplay.^7–9^ This comprehensive review aims to synthesize the latest knowledge on eosinophil metabolism; unravel the intricate relationship between eosinophils, adipocytes, and host metabolism; and explore the implications of these interactions in both health and disease.

## 2. Part 1: impact of eosinophils on adipocytes

### 2.1 Mechanisms of eosinophil–adipocyte interactions

Eosinophils can interact with adipocytes in adipose tissue, both indirectly and directly. Adipose tissue houses a diverse population of immune cells, including macrophages, lymphocytes, and eosinophils.^10^ Consequently, eosinophils can engage with adipocytes indirectly through intermediary white blood cells. Metabolically activated macrophages in obese adipose tissue contribute to metabolic dysfunction and insulin resistance, as shown in human and animal studies.^11–13^ Adipose eosinophils, as the primary source of interleukin (IL) 4, communicate with macrophages and polarize macrophage function.^14^ In murine adipose tissue, the IL-4 interaction between eosinophils and macrophages leads to the activation of uncoupling protein 1, a mitochondrial protein devoted to adaptive thermogenesis, which promotes beiging of white adipose tissue and enhances thermogenesis.^14^

The indirect interaction between eosinophils and adipocytes may also involve neuronal control. Adipose tissue is highly innervated by the sympathetic nervous system. While most peripheral tissues receive dual innervation from both branches of the autonomic nervous system, the sympathetic nervous system (SNS), and the parasympathetic nervous system (PSNS), the presence of PSNS innervation in white adipose tissue (WAT) is controversial.^15–17^ The degree of SNS innervation also varies in the tissue under different metabolic states. Animal studies indicate that cold stress or prolonged fasting can increase sympathetic innervation in adipose tissue.^18,19^ In cold stress, eosinophils in adipose tissue produce more nerve growth factor (NGF).^20^ NGF can stimulate sympathetic axonal outgrowth and activation, leading to the release of norepinephrine (NE) in adipose tissue, which signals to adipocytes, inducing lipolysis and conversion of WAT to brown adipose tissue.^21^ NE also can serve as positive feedback, promoting the release of IL-33 and the activation of type 2 innate lymphoid cells (ILC2s) in adipose tissue.^20^ These ILC2s subsequently secrete IL-5 to recruit eosinophils, which in turn release more NGF to further promote the sympathetic innervation in the adipose tissue.^20^

Mechanisms of the direct interaction between adipocytes and eosinophils are currently underexplored. Both animal and human studies suggest that eosinophils can directly modulate adipocyte differentiation and function, which was also emphasized in the commentary by Jacobsen and De Filippis.^7,22–24^ In particular, recent animal research indicates that eosinophils may directly facilitate the “browning” of WAT via changing adipocyte transcriptional responses, exemplified by Krüppel-like factor 3, which transcriptionally regulates adipocyte beiging.^22^ Moreover, adipocytes express hormone fibroblast growth factor 21 (FGF21), which induces the production of the eotaxin C-C motif chemokine ligand 11 (CCL11) to promote the recruitment of eosinophils into adipose tissue.^25,26^ In turn, adipose-recruited eosinophils may trigger and promote beiging in adipose tissue. Furthermore, as shown in both animal and human studies, eosinophils are proficient in secreting various cytokines and chemokines, such as IL-4 and IL-13, which may directly influence behavior of adipocytes and other immune cells resident in adipose tissue, ultimately influencing systemic metabolism.^24,27,28^

Eosinophil participation in lipolytic processes of adipose tissue is another poorly understood aspect of adipose regulation. Lipolysis accompanied by activation of arachidonic acid metabolism is an essential process of fat breakdown in the regulation of insulin sensitivity and systemic energy metabolism.^29^ Activation of the FGF21 axis in animals regulates lipolysis in WAT, therefore directly implicating eosinophils in this process.^30,31^ Moreover, human eosinophils are known to constitutively and abundantly express eoxins, such as 15 lipoxygenase 1 (15LO1) (ALOX15), which could be one way adipose eosinophils participate in lipolytic process.^32^ Other, albeit unexplored, mechanisms could involve production of adipose stromal remodeling factors (matrix metalloproteinases, growth factors, granular proteins) by eosinophils to guide adipocyte differentiation or a direct expression of enzymes regulatory for lipolysis. However, the exact phenotype and function of adipose eosinophil subsets is not well characterized, which obscures the nature of the direct interactions between adipose eosinophils and adipocytes and necessitates future research.

### 2.2 Protective effects of eosinophils in obesity

Eosinophils play diverse roles in various tissues, contributing to both normal physiological functions and responses to diseases. Traditionally associated with parasitic infections and allergic responses, eosinophils have recently been discovered as unexpected residents within adipose tissue.^1–4,7–10^ Within this microenvironment, eosinophils exhibit an obesity-resistant effect, as evidenced by both animal and human data, while also serving dual roles as proinflammatory and anti-inflammatory factors, depending on the context. A recent human study highlights the significant reduction in adipose tissue eosinophil content among obese patients.^24^ This finding underscores the obesity-resistant effect of eosinophils within this microenvironment. Notably, the protective effect of eosinophils against high-fat diet-induced obesity in mice was first reported in 2011 by Wu et al.^9^ Their study demonstrated that C57BL/6 mice lacking eosinophils (ΔdblGATA mice) and fed a high-fat diet exhibited increased body fat, impaired glucose tolerance, and decreased insulin sensitivity compared to their wild-type littermate control mice. Conversely, increasing eosinophil levels may prevent weight gain. For instance, chronic helminth infection or the administration of soluble helminth egg antigens can increase eosinophil levels in high-fat diet-induced obese mice. Consequently, the treated mice show reduced weight gain, decreased fat mass gain, smaller adipocyte size, improved peripheral glucose uptake, and enhanced insulin sensitivity in WAT.^33,34^ In another study, honeybee pollen extract was used to increase eosinophils in mesenteric and epididymal adipose tissue of ob/ob mice, which develop obesity and insulin resistance due to a mutation in the leptin gene. The increased adipose eosinophils restored glucose tolerance and insulin sensitivity in pollen-treated mice.^35^ These animal studies consistently confirm that adipose eosinophils play a crucial role in regulation of systemic metabolic homeostasis via interactions with adipocytes and/or adipose leukocytes.^33–35^ However, it is noteworthy that an acute increase in adipose eosinophils achieved through short-term treatment with helminth antigens does not protect against diet-induced obesity, indicating that the effectiveness of this protective mechanism requires sustained interactions over an extended time period.^36^

### 2.3 Eosinophil–adipose interactions in the contexts of metabolic health and obesity

The universal protective effect of eosinophils in obesity is still under debate. Some studies report positive regulation of adipose function and weight loss in mice due to eosinophils, while others report no change in metabolic impairment. Findings of animal studies conclude that eosinophils may influence adipose metabolism in healthy conditions but do not improve adipose function in metabolic dysregulation.^33,34,37^ The variations in these studies may arise from differences in the recovery of eosinophil numbers and alterations in the properties of adipose eosinophils themselves following systemic metabolic shifts. Studies restoring eosinophil number using IL-5 intraperitoneal injections do not show any impact on reversing adipose dysfunction. In the state of obesity or metabolic disorders, adipocytes release more leptin, which may change the phenotype of adipose eosinophils.^38,39^ While IL-5 injection likely increases maturation of eosinophil precursors and their release from the bone marrow, once those mature cells enter circulation and traffic to the adipose tissue, leptin activation may skew them toward a prosurvival and proinflammatory phenotype.^40–42^ The influence that leptin and other adipokines have on eosinophil phenotype could also contribute to comorbidities associated with obesity.

**Fig. 1.**
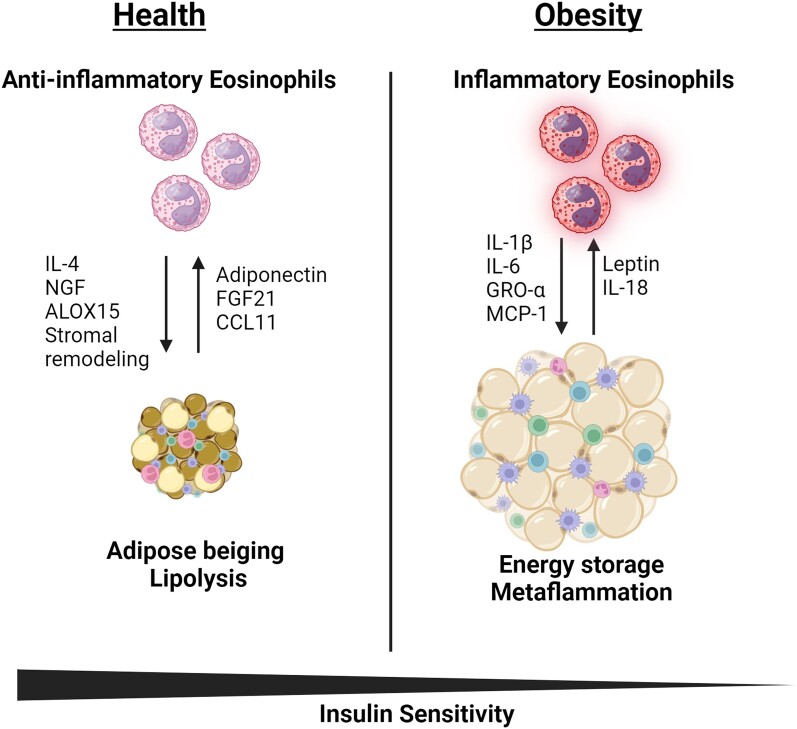
Adipose eosinophils exhibit phenotypic and functional changes based on the metabolic status of the host. Adipose eosinophil phenotype is regulated by the fat tissue microenvironment, including key adipokines. Adipokines such as adiponectin, FGF21, and CCL11 maintain adipose eosinophils in an anti-inflammatory state. Conversely, adipokines such as leptin and IL-18 induce a phenotypic shift toward inflammatory eosinophils. Under healthy metabolic conditions, adipose eosinophils adopt an anti-inflammatory phenotype, producing cytokines and growth factors like IL-4, NGF, and ALOX15, which promote stromal remodeling, adipose beiging, lipolysis, and increased insulin sensitivity, potentially mitigating obesity. In a metabolic dysregulation state such as in obesity, adipose eosinophils shift to a proinflammatory phenotype, releasing cytokines such as IL-1β, IL-6, GRO-α, and MCP-1, promoting adipose tissue expansion, insulin resistance, and energy storage. Although type 2 mediators can be seen as proinflammatory in contexts of allergy, type 2 response plays a beneficial, anti-inflammatory role in the adipose tissue.

If eosinophils were restored in mice on a high-fat diet through a mechanism that matures and activates them independently of leptin, these eosinophils could retain homeostatic functions. This would explain why chronic helminth infection or exposure to soluble helminth egg antigen led to increased adipose tissue eosinophils, resulting in reduced mouse overall weight and fat mass while increasing glucose uptake and insulin sensitivity.^33,34^ Helminth infection initiates activation and survival of human or animal eosinophils, leading to an eosinophil phenotype (i.e. anti-inflammatory).^43,44^ Helminth infection in mice would induce upregulation of serum IL-4, which may counter the proinflammatory environment and regulate homeostatic functions.^14,43^ The phenotype of the resulting eosinophils will be anti-inflammatory in the context of adipose homeostasis as they would preferentially produce cytokines to support an anti-inflammatory adipose tissue microenvironment and lipolysis (Fig. 1).^27,28,36^

In summary, eosinophils appear to play a role in maintaining adipose tissue homeostasis. They may contribute to the regulation of adipocyte size, adipokine secretion, and overall adipose tissue metabolism. However, the nature of the intricate crosstalk between eosinophils and adipocytes may be dependent on host metabolic state, which needs to be understood further if we become interested in harnessing these interactions for therapeutic purposes (Fig. 1).

## 3. Part 2: metabolic regulation of eosinophil

### 3.1 Metabolic dysregulation results in metabolic shift in eosinophils

There are a number of studies investigating how eosinophils influence the host's metabolism and adipose tissue function. However, understanding how metabolic dysfunction in the host influences eosinophil biology and phenotype remains limited. Metabolic disorders, which feature chronic hypoxia, insulin resistance, and poor glucose uptake, prompt distinct response in various cells.^45–48^ For instance, Peters et al.^47^ examined homeostatic model assessment for insulin resistance (HOMA-IR) values and lung function in patients with severe asthma. They discovered that insulin resistance is common in asthma and is associated with lower lung function, as well as suboptimal responses to bronchodilator and corticosteroid treatments.

Eosinophils exhibit remarkable metabolic flexibility, enabling them to adapt effectively to diverse microenvironments.^48^ Human eosinophils have shown the capacity to switch between glycolysis, glucose oxidation, and mitochondrial oxidative phosphorylation in response to environmental demands, a feature not shared by their close relative, neutrophils. Nevertheless, it remains unknown how these metabolic switches in eosinophils affect their phenotype and roles in host defense, homeostasis, and inflammation. Based on current knowledge, it is plausible that the microenvironment fostered by metabolic dysregulation potentially induces a prosurvival and proinflammatory phenotype in eosinophils.

Obesity and diabetes induce a chronic hypoxic state that could initiate a shift in eosinophil metabolism and phenotype. Hypoxia is a potent proinflammatory stimulus for human peripheral blood eosinophils, upregulating prosurvival and proangiogenic functions.^49^ Under hypoxic conditions, human eosinophils undergo a metabolic shift from mitochondrial oxidative phosphorylation to glycolysis.^48^ Eosinophils increase the expression of GLUT1 under hypoxic conditions and activate hypoxia-inducible factor 1α (HIF-1α) signaling. HIF-1α signaling decreases mitochondrial metabolism while upregulating the production of glycolytic enzymes and glucose transporters.^50,51^ This metabolic shift allows both human and animal eosinophils to remain active in inflammatory environments. Furthermore, hypoxia enhanced human eosinophil secretion of Charcot–Leyden crystals, which are highly proinflammatory, while delaying constitutive apoptosis.^51^ This indicates that the shift from mitochondrial oxidative phosphorylation to glycolysis induces prosurvival and proinflammatory phenotype.

Similarly, our work shows that subjecting bone marrow–derived murine eosinophils to glucose deprivation in the process of hematopoietic differentiation results in a long-lived phenotype in vitro.^45^ These murine eosinophils, derived from bone marrow, exhibit increased metabolic flexibility when faced with sustained glucose starvation. They transition from relying on glucose as their primary energy source to utilizing amino acids and fatty acids. Remarkably, after adapting to a glucose-free environment, these eosinophils persist in culture for more than 40 d, indicating that a metabolic shift away from glucose energetic consumption could potentially lead to a prosurvival phenotype. Glucose starvation is another feature of metabolic dysregulation, often associated with insulin resistance. There is still a lot more work to be done in comprehending how changes in energy metabolism influence eosinophil biology and phenotype.

### 3.2 Adipose tissue–derived adipokines regulate eosinophil phenotype

In addition to its traditional role as a storage depot for excess energy in the form of triglycerides, adipose tissue serves as a dynamic immune microenvironment. Adipose tissue has the capacity to produce a wide array of regulatory molecules, such as cytokines, chemokines, and adipokines. Adipokines, released by adipocytes and adipose macrophages in response to an increase in fat mass, not only improve eosinophil survival but also promote production of inflammatory cytokines, induce migration, and alter expression of cell surface receptors on eosinophils (Fig. 1).^38–42^

Leptin, a key hormone in energy homeostasis and one of the most studied adipokines, exerts control over appetite and energy expenditure by signaling in the hypothalamus. Metabolic dysregulation often leads to leptin resistance, resulting in an increase in circulating leptin as a compensatory effect.^39^ Human eosinophils have been shown to express leptin receptor Ob-Rb (LEPR) and exhibit a dose-dependent migration toward leptin, which amplifies their chemotactic response to eotaxin in vitro.^40^ Leptin-induced eosinophil migration is associated with mitogen-activated protein kinase (MAPK) activation. In vivo, leptin has also been shown to induce mouse eosinophil migration and activation directly and indirectly.^41^ Leptin signaling occurs directly via the Ob-Rb leptin receptor and indirectly via mast cell–mediated production of CCL5 and PGD2, which act on eosinophil receptors CCR3, DP1, and DP2.^41^ Beyond migration, leptin also regulates the expression of cell surface receptors and proinflammatory cytokines in eosinophils. Leptin has been shown to upregulate expression of intercellular adhesion molecule 1 (ICAM-1) and CD18 on human eosinophils, which may aid in the recruitment of eosinophils to inflammatory sites and their transmigration.^42^ Interestingly, in the same study, leptin downregulated the expression of ICAM-3 and L-selectin, both of which have anti-inflammatory functions and are present in resting eosinophils before activation.^52,53^ This study suggests that leptin not only initiates eosinophil transmigration and activation but also transforms eosinophils into a proinflammatory phenotype. Leptin was also found to selectively induce the synthesis and release of proinflammatory cytokines, including IL-1β, IL-6, IL-18, growth-regulated protein alpha (GRO-α or CXCL1), and monocyte chemoattractant proten-1 (MCP-1), aligning with the conclusion that leptin converts human eosinophils into a proinflammatory phenotype.^42^

As leptin levels rise in the context of metabolic dysregulation, adiponectin levels decline. Adiponectin, an adipokine secreted by adipose tissue, opposes the actions of leptin by increasing insulin sensitivity and exerting anti-inflammatory actions.^54^ Particularly, a decrease in the adiponectin/leptin ratio is correlated with insulin resistance and adipose tissue dysfunction.^55^ During the development of metabolic diseases, plasma adiponectin concentration decreases,^56^ while leptin concentration increases due to the development of leptin resistance. Human eosinophils express leptin receptors but also adiponectin receptors (AdipoR1 and AdipoR2).^57^ Given the conflicting relationship between leptin and adiponectin, it would not be surprising that they may also have opposing effects on eosinophil biology. Indeed, it has been demonstrated that adiponectin inhibits human eosinophil eotaxin-enhanced adhesion as well as eotaxin-directed chemotactic responses.^57^

In summary, the systemic microenvironmental changes that occur during metabolic dysregulation, such as impaired glucose uptake, hypoxia, and the increased presence of proinflammatory adipokines, lead to the development of prosurvival and proinflammatory phenotypes in eosinophils (Fig. 1). The altered eosinophils likely contribute to the overall systemic proinflammatory environment. In other words, the influence of the microenvironment on eosinophils acts as a positive feedback loop to maintain the chronic proinflammatory environment of metabolic dysregulation.

## 4. Part 3: host metabolism–eosinophil interactions in obese vs allergic asthma

### 4.1 Granulocyte responses in obesity-related asthma

By 2030, more than 50% of the US population is projected to become obese.^58^ Obesity, a major risk factor for a range of health conditions, including asthma, increases the prevalence, incidence, and severity of asthma while compromising asthma control.^59,60^ This establishes obesity-related asthma as a complex and increasingly prevalent clinical endotype within the broader spectrum of asthma. Some obese patients with asthma have severe asthma symptoms with low sputum eosinophils while others have high eosinophils.^61,62^ Thus, the obesity-related asthma phenotype can be divided into at least 2 subclasses: Th2 high-obese patients, characterized by preexisting allergic asthma, with high eosinophils additionally complicated by obesity, vs Th2 low-obese patients with asthma who develop asthma symptoms as a consequence of obesity and have lower eosinophils and mixed granulocyte responses.^63^ For Th2 low-obese asthmatic patients, obesity may cause airway hyperresponsiveness through an eosinophil-independent mechanism, including insulin resistance and subsequent hyperinsulinemia.^64–72^ Recent human studies, such as the one conducted by Peters et al.,^47^ have corroborated findings from animal research, demonstrating that insulin resistance is associated with reduced lung function and suboptimal responses to bronchodilator and corticosteroid treatments in asthma patients. In general, Th2 high asthma is referred to as “eosinophilic,” and Th2 low asthma frequently features a mixed granulocytic or paucigranulocytic phenotype.^73^ These observed differences between subphenotypes of obesity-related asthma suggest that host metabolic processes may regulate the nature of granulocyte responses.

Clinical evidence regarding the protective role of eosinophils in obesity remains inconclusive and requires further investigation due to limited human studies and conflicting results. Eosinophilia can coexist with obesity, as several human studies have demonstrated a positive correlation between blood eosinophil counts and body mass index (BMI) or metabolic syndrome.^74–77^ Sunadome et al.^76^ found a positive correlation between blood eosinophils and BMI, but this correlation switched to negative for patients with the highest blood eosinophils counts. Notably, these small-size clinical studies only include cell count of blood eosinophils, but not adipose eosinophils. This omission is significant, considering recent human studies have shown that specifically adipose tissue eosinophils have more influence on weight gain and metabolic dysfunction in obesity.^24,77^ Kuruvilla et al.^78^ observed a mild but significant decrease in BMI of severe asthma patients over 6 mo on anti–IL-5 therapy. Again, eosinophils were only measured in blood, not in body fat. It is crucial to note that this study lacked randomization, placebo control, and other obesity-related measurements such as body fat and glucose tolerance.^78^ Considering that severe asthmatic patients often use corticosteroids and have limited exercise capacity due to asthma complications, this may contribute to weight gain independent of eosinophils. Thus, the observed decrease in body weight during anti–IL-5 or anti–IL-5 receptor biologic treatment might be linked to reduced steroid use or increased physical activity.

### 4.2 Host metabolic dysregulation in obese asthma may directly impact eosinophil development and function

It is important to understand that obesity is a systemic process that affects metabolism and development of all cells, including eosinophils. A survey of the literature reveals that the microenvironment fostered by metabolic disorders predominantly promotes the inflammatory subset of human and animal eosinophils.^40–42,45–48^ For instance, a human data analysis shows that a subset of obese asthma patients has high levels of IL-6, which is an important cytokine mediating the interaction between adipose cell and eosinophils.^79^ The fate of tissue-resident eosinophils that perform homeostatic functions, such as tissue repair, remodeling, epithelial structure maintenance, and regulation of adipose tissue metabolism, remains a critical question. Contrary to proinflammatory eosinophils, these homeostatic eosinophils have not been reported to be expanded during metabolic dysregulation; instead, they are reported to be depleted in various tissues. For instance, tissue-resident intestinal eosinophils, contributing to gut immune homeostasis, tissue integrity, epithelial repair, and barrier function in homeostasis, were found to be depleted in mice maintained on high-fat diet.^80,81^ Eosinophil depletion in high-fat diet-fed mice models was associated with ileal paracellular permeability. Similarly, studies have consistently demonstrated a reduction of tissue-resident adipose eosinophils in mice fed a high-fat diet as well as mice with genetic obesity secondary to leptin deficiency (ob/ob), although this may be related to an overall increase in the adipocyte/eosinophil ratio associated with an overall increase in adipose tissue mass.^7,9,33,37^

Furthermore, it is important to understand that metabolism controls hematopoiesis of immune cells, including eosinophils. Factors such as insulin sensitivity, mitochondrial activity, and the ability to switch between metabolic pathways during different stages of hematopoiesis are critical in determining the final phenotype and function of granulocyte.^82,83^ Eosinophils upregulate energy glycolysis during maturation, and different subsets of eosinophils may require different levels and nutritional sources of energy. Disruption of normal metabolic processes in the obese host may result in functionally different cell subsets or affect the ratios of different granulocytes due to improper metabolic regulation of hematopoiesis. This may underpin observations such as changes from eosinophilic Th2 high to mixed Th2 low granulocyte responses in obese asthma, or an increased prevalence of inflammatory over remodeling subsets of eosinophils, which warrants further mechanistic examination.

### 4.3 Metaflammation and airway hyperresponsiveness

Another key aspect to consider is the proinflammatory environment associated with obesity. Adipose tissue in obese individuals exhibits chronic low-grade inflammation, often referred to as metaflammation.^84^ This inflammatory milieu extends to the respiratory system, impacting airway function.^85^ Adipocytes themselves are a source of factors that can directly influence asthma pathophysiology. Adipokines, which we discussed in section 2, such as leptin, resistin, and adiponectin, have been implicated in modulating airway inflammation and bronchial reactivity. For instance, leptin, an adipokine that regulates appetite and energy expenditure, has been shown to promote airway inflammation and hyperresponsiveness. Conversely, adiponectin, with its anti-inflammatory properties, may have a protective effect on the airways. These findings underscore the multifaceted role of adipocytes in shaping the asthma phenotype in obesity.

### 4.4 Implications for human diseases

The evidence we show in this review on how the host's metabolic status influences the phenotype of eosinophils present in the adipose tissue is largely based on animal studies or ex vivo experiments involving isolated human eosinophils. This is as a result of the scarcity of human studies addressing eosinophil biology in patients with metabolic syndromes, which is a significant gap in the field. Based on available human studies, we can extrapolate that human adipose eosinophils undergo similar processes as mouse adipose eosinophils in responding to changes in the host's metabolic status and possibly modulate obesity in their anti-inflammatory status. We do know that humans and mice share similar metabolic processes within the adipose tissue eosinophils.^86,87^ Oliveira et al.^8^ demonstrated that human eosinophils seem to modulate glucose homeostasis within the adipose tissue based on correlation of homeostatic eosinophil markers and insulin sensitivity markers. Human eosinophils also have the capacity to respond to microenvironmental changes (such as hypoxia) and participate in signal transduction with adipokines within the adipose tissue.^40,42,49,57^ Human eosinophils have been shown to undergo a metabolic shift in response to microenvironmental changes.^48^ Based on this, it is possible that human adipose eosinophils might be similar to mouse adipose eosinophils in responding to metabolic change. Clearly, there is an urgent need to understand the role of eosinophils in human adipose tissue in order to understand metabolic health better and explore the possibility of eosinophil-based treatments for metabolic disorders and obesity-related asthma.

## 5. Summary

Taken together, all the studies discussed above highlight the significance of host metabolism in determining eosinophil–adipose interactions. We believe investigating the microenvironmental factors—metabolic, inflammatory, epigenetic—that give rise to eosinophil heterogeneity within various tissue contexts is truly the key to understanding how metabolic dysregulation impacts eosinophil biology. Understanding the intricate interactions between eosinophils, adipocytes, and metabolism has profound implications for therapeutic strategies targeting obesity-related asthma. Therapies that address both metabolic dysfunction and airway inflammation may hold promise in managing this complex phenotype. Moreover, personalized approaches considering an individual's adipose tissue biology, eosinophilic profile, and metabolic status may lead to more effective interventions. As research in this field advances, novel therapeutic targets are likely to emerge, offering new avenues for precision medicine in the treatment of metabolic disorders.

## 6. Conclusions and future directions

Metabolism remains the least studied and understood aspect of eosinophil biology, which we currently see as the newest frontier in the field. Our review delves into the intricate interplay between eosinophils, adipocytes, and host metabolism, providing additional insights into their systemic roles in regulation of metabolic homeostasis. Traditionally viewed as immune responders in allergies, eosinophils now emerge as influential players shaping adipocyte function and influencing systemic metabolism. While recent studies have provided valuable insights into the crosstalk between eosinophils and adipocytes, many mechanistic details remain to be elucidated. Further investigations, particularly in human studies, are needed to unravel the precise cellular and signaling interactions, cytokines, and chemokines that govern this dialogue. Direct regulation of adipocytes by eosinophils, distinct from regulation via macrophage intermediates, is currently underappreciated as a fundamental mechanism in adipose tissue regulation. A more comprehensive understanding of these mechanisms holds the potential to unveil the untapped therapeutic opportunities.

Excitingly, identifying biomarkers reflecting the eosinophil–adipocyte interplay could revolutionize risk stratification for metabolic disorders and guide personalized treatment strategies. Translating this knowledge into therapeutic interventions is a promising avenue, targeting eosinophilic inflammation and metabolic dysregulation for conditions such as obesity-related asthma. Exploring the long-term health outcomes associated with eosinophil–adipocyte interactions in the contexts of obesity, diabetes, and associated inflammatory disorders is pivotal for shaping effective public health strategies.

In conclusion, our review provides a snapshot of current knowledge on eosinophils, adipocytes, and host metabolism. Ongoing research promises to unravel the intricacies of this relationship in health and disease, offering innovative diagnostic tools and targeted therapies to enhance the lives of those affected by metabolic disorders.
